# The Potential of Epigallocatechin-3-gallate (EGCG) as Complementary Medicine for the Treatment of Inflammatory Bowel Disease

**DOI:** 10.3390/ph16050748

**Published:** 2023-05-14

**Authors:** Sabrina Schnur, Fabian Hans, Annika Dehne, Janina Osti, Malte-Ole Schneemann, Marc Schneider, Marius Hittinger

**Affiliations:** 1 Department of Pharmacy, Biopharmaceutics and Pharmaceutical Technology, Saarland University, 66123 Saarbrücken, Germany; sabrina.schnur@uni-saarland.de (S.S.); marc.schneider@uni-saarland.de (M.S.); 2 Department of Drug Delivery, PharmBioTec Research and Development GmbH, 66123 Saarbrücken, Germany; 3 3RProducts Marius Hittinger, 6640 Blieskastel, Germany

**Keywords:** intestinal barrier integrity, transepithelial electrical resistance, pro-inflammatory cytokines, monocyte-derived macrophages, natural product, green tea, antioxidant

## Abstract

Complementary and alternative medicine has the potential to enrich conventional therapy to improve the treatment of various diseases. Patients that suffer from inflammatory bowel disease, which requires a constant need for medication, have to deal with the adverse effects of repeated application. Natural products such as Epigallocatechin-3-gallate (EGCG) possess the potential to improve symptoms of inflammatory diseases. We investigated the efficacy of EGCG on an inflamed co-culture model simulating IBD and compared it to the efficacies of four commonly applied active pharmaceutical ingredients. EGCG (200 µg/mL) strongly stabilized the TEER value of the inflamed epithelial barrier to 165.7 ± 4.6% after 4 h. Moreover, the full barrier integrity was maintained even after 48 h. This corresponds to the immunosuppressant 6-Mercaptopurin and the biological drug Infliximab. The EGCG treatment significantly decreased the release of the pro-inflammatory cytokines IL-6 (to 0%) and IL-8 (to 14.2%), similar to the effect of the corticosteroid Prednisolone. Therefore, EGCG has a high potential to be deployed as complementary medicine in IBD. In future studies, the improvement of EGCG stability is a key factor in increasing the bioavailability in vivo and fully harnessing the health-improving effects of EGCG.

## 1. Introduction

The interest in traditional, complementary, and alternative medicine (T/CAM) has increased over the past decades [1]. Here, “traditional medicine” describes indigenous health traditions, and the term “complementary medicine” is defined as a combination of a non-mainstream approach with conventional medicine, such as nutritional supplements (e.g., natural products) or manual therapies. “Alternative medicine” refers to the usage of these approaches instead of conventional medicine [2,3]. The National Center for Complementary and Integrative Health (NCCIH) focuses on the integration of evidence-based complementary medicine in conventional therapy, as recently addressed in the NCCIH Strategic Plan FY 2021–2025 [4]. Furthermore, the World Health Organisation (WHO) states in their WHO Traditional Medicine Strategy 2014–2023 that the development of norms and standards based on reliable data for T/CAM to safely integrate qualified and effective therapies in the health systems of the WHO Member States [5]. Thus, it is evident that reliable and meaningful data for potential T/CAM therapies are required. One considerable motivation of the general public to utilize T/CAM in their daily life is the potential prevention and management of chronic, often lifestyle-related, diseases [1]. A prominent example is patients that suffer from Inflammatory Bowel Disease (IBD) [6,7]. IBD is defined by chronic inflammations of the gastrointestinal (GI) tract that are subdivided into two main types of the disease: crohn’s disease (CD) and ulcerative colitis (UC) [8]. Numerous patients complement their conventional treatment of the symptoms with T/CAM applications, mostly herbal therapies [6]. In this context, Epigallocatechin-3-gallate (EGCG) is a promising natural product for complementary therapy of chronic inflammations [9].

EGCG is the most abundant polyphenol in green tea extract (GTE) and a strong antioxidant, which is reported to provide numerous health benefits, such as anti-inflammatory, anti-bacterial, and anti-cancerogenic effects [10]. With respect to IBD, the efficacy of EGCG was shown by using various animal models mimicking UC or CD [11,12,13,14,15]. For instance, EGCG significantly improved disrupted colon architecture and leukocyte infiltration and reduced the level of the pro-inflammatory cytokine tumor necrosis factor-alpha (TNF-α) in a dinitrobenzene sulphonic acid (DNBS) rat model. Moreover, disease-related symptoms such as diarrhea and weight loss were improved [11]. The efficacy of EGCG for the treatment of IBD was further evaluated by comparison with the active pharmaceutical ingredient (API) sulfasalazine that was applied to treat an acetic acid-induced colitis rat model or a dextran sodium sulfate (DSS) mouse model. Analogous effects for EGCG and sulfasalazine were observed, such as a decrease in the disease activity index (DAI) and histological score, as well as the reduction of TNF-α, INF-γ, and the nuclear factor-κBp65 (NF- κBp65) serum levels [12,13]. Recently published studies underline the potential of EGCG for the treatment of IBD in animal studies [14,15]. However, the transfer of the EGCG data to the human condition remains an evident challenge [16]. One reason is the low bioavailability and stability of EGCG at physiological conditions due to its chemical structure [16,17,18] and physiological metabolic biotransformation [19,20,21]. The biotransformation, such as methylation or glucuronidation of EGCG, is reported to be highly dependent on the tissue and the species [19]. Further open questions that need to be addressed are the dose-finding as well as the time and frequency of administration [16]. The most prominent challenge is the translation of the EGCG in vitro data to the human in vivo situation to achieve a non-toxic, effective dose [22]. Although several clinical trials were performed and are still ongoing, EGCG has not been approved for medical usage yet and showed in some clinical trials a rather poor efficacy combined with a low plasma concentration [10,16,22]. Therefore, the investigation of EGCG efficacy using human-based, reliable, and predictive in vitro models that simulate different tissues and diseases for pre-clinical assessment are required to improve the limitations of EGCG.

In this study, we focused on the investigation of the potential of EGCG for the treatment of IBD, simulated by a recently established human-cell-based inflammable co-culture to mimic the application of EGCG to humans. The co-culture consists of epithelial cells and primary immune cells to address the role of both: the intestinal barrier and the immune cells in IBD, including donor dependency on the immune system. In our previous work, we evaluated the predictivity of our in vitro model by the treatment of the induced inflammation with commonly applied active pharmaceutical ingredients (APIs) related to the severity level of the disease [23]. We identified the transepithelial electrical resistance (TEER) as the most predictive readout, which facilitates the investigation of the epithelial barrier integrity in IBD. Additionally, the mode of action-dependent effect on the reduction of pro-inflammatory cytokines can be analyzed [23]. We aimed for the comparison of the EGCG efficacy with the efficacies of commonly applied APIs in IBD related to four different severity levels: from minor inflammations up to major inflammations. The investigation of EGCG on the same in vitro model in an equipotent concentration enables both: the assessment of the general efficacy and the evaluation of the efficacy level (minor up to strong) on two disease-related key compartments: the intestinal barrier and the immune system. The results might augment the database for T/CAM integrating EGCG as a promising complementary medicine. The presented in vitro model provides the possibility to investigate improved formulations of EGCG for the treatment of IBD with respect to stability enhancement or increased bioavailability to address limitations in vivo. In our experimental setup, we tested EGCG at the two concentrations 2 µg/mL and 200 µg/mL. EGCG was applied to the immune cells located in the basolateral compartment after the co-culture was inflamed. The integrity of the epithelial barrier was determined over 48 h by TEER measurements. In addition, the effect of EGCG on the immune cells was investigated by analysis of the cytokine release. The cell viability of the epithelial and immune cells was determined to investigate possible cytotoxic effects during the treatment. In a next step, the effect of EGCG on the non-inflamed intestinal Caco-2 barrier was analyzed in more detail. Finally, we compared our results for EGCG with the efficacies of the APIs currently used in the treatment of IBD.

## 2. Results

### 2.1. EGCG Treatment of the Inflamed Co-Culture

**Figure 1:**
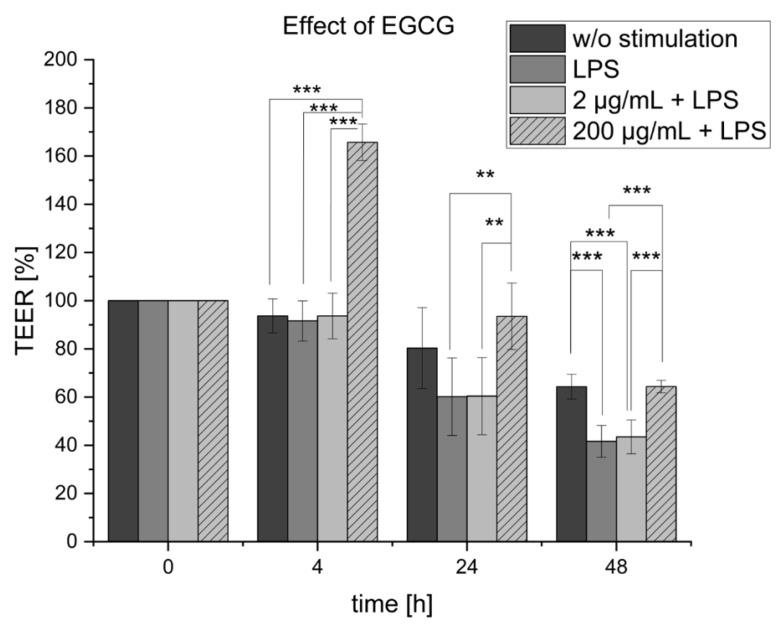
Effect of 2 µg/mL and 200 µg/mL Epigallocatechin-3-gallate (EGCG) over time on the lipopolysaccharides (LPS)-inflamed co-culture determined by transepithelial electrical resistance (TEER) measurements of the Caco-2 cells located in the apical compartment. The co-culture without any stimulation served as negative control (no inflammation = healthy state), and the LPS-inflamed co-culture was utilized as a positive control (inflammation = disease state). Results are displayed as mean ± standard deviation for in summary n = 9 wells for each group based on three biological replicates. The *p*-values ** *p* < 0.01, and *** *p* < 0.001 imply significant differences.

The potential anti-inflammatory effect of EGCG of the two concentrations, 200 µg/mL and 2 µg/mL, was investigated. EGCG was applied to the lipopolysaccharides (LPS) stimulated monocyte-derived macrophages (MDM) in the basolateral compartment of the co-culture setup. Figure 1 shows the effect on the barrier properties of the Caco-2 cells located in the apical compartment determined by TEER measurements after 4 h, 24 h, and 48 h. The application of 2 µg/mL EGCG showed no measurable positive effect on the TEER values and was equal to the LPS control. However, the treatment with EGCG in the concentration of 200 µg/mL resulted in a significant increase in the TEER values up to 165.7 ± 4.6% after 4 h in comparison to the LPS-inflamed co-culture (91.6 ± 9.1%) and even to the co-culture without any stimulation (healthy state). After 24 h and 48 h, the increase in TEER in comparison to the LPS-inflamed group was still significant; however, it was decreasing over time and not as high as the TEER value measured after 4 h. Nevertheless, the EGCG treatment led to a regain of healthy barrier properties by restoring the TEER values to the level of the healthy co-culture.

### 2.2. Effect of EGCG on Cytokine Release of MDM

**Figure 2:**
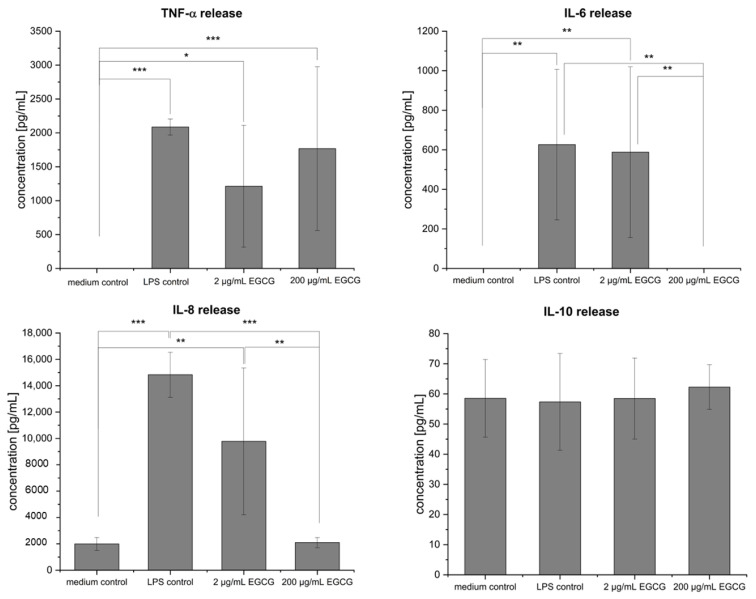
Effect of LPS stimulation, 2 µg/mL, and 200 µg/mL EGCG treatment on the cytokines released by the MDM in the co-culture setup in comparison to the healthy state (medium control). The stimulation with LPS led to an increase in the tumor necrosis factor-alpha (TNF-α), Interleukin (IL)-6, and IL-8 release. No significant effect of the treatment with 2 µg/mL EGCG was observed. After the treatment with 200 µg/mL EGCG, no IL-6 release was detectable, and a significant decrease in the IL-8 release was achieved. Results are displayed as mean ± standard deviation for in summary n = 9 wells for each group (except for LPS (TNF-α) *n* = 6 wells). Samples out of three biological replicates were utilized. * *p* < 0.05, ** *p* < 0.01, and *** *p* < 0.001 imply a significant difference.

Following the stimulation and treatment of the co-culture, the cytokine release from the MDM located in the basolateral compartment was investigated (Figure 2). In the healthy state, no TNF-α was secreted; whereas the stimulation with LPS resulted in a TNF-α release of 2086.9 ± 118.42 pg/mL. The treatment of the inflamed co-culture with 2 µg/mL EGCG and 200 µg/mL EGCG led to no significant changes (1212.3 ± 899.95 pg/mL for 2 µg/mL and 1767.7 ± 1207.94 pg/mL for 200 µg/mL EGCG) of the TNF-α release compared to the inflammation control. A significant increase in the release after LPS stimulation (to 626.5 ± 380.36 pg/mL) was additionally observed for IL-6. Similar to the TNF-α release, no effect on the IL-6 release after the treatment with 2 µg/mL EGCG (588 ± 431.54 pg/mL) was measurable; however, there was a significant change to the release of IL-6, which was undetected after the treatment with 200 µg/mL EGCG. The stimulation with LPS led to a significant increase of the IL-8 release (to 14,831.9 ± 1702.29 pg/mL) in comparison to the medium control (1994.7 ± 488.43 pg/mL). While the treatment of the LPS-inflamed co-culture with 2 µg/mL EGCG (9778.6 ± 5566.65 pg/mL) had no effect on the IL-8 release, a significant effect for 200 µg/mL EGCG (decrease to 2100.6 ± 384.19 pg/mL) was observed. For the anti-inflammatory cytokine IL-10, only a lower level of around 60 pg/mL was measured for all groups.

### 2.3. Cell Viability of the Co-Culture Setup after EGCG Treatment

**Figure 3:**
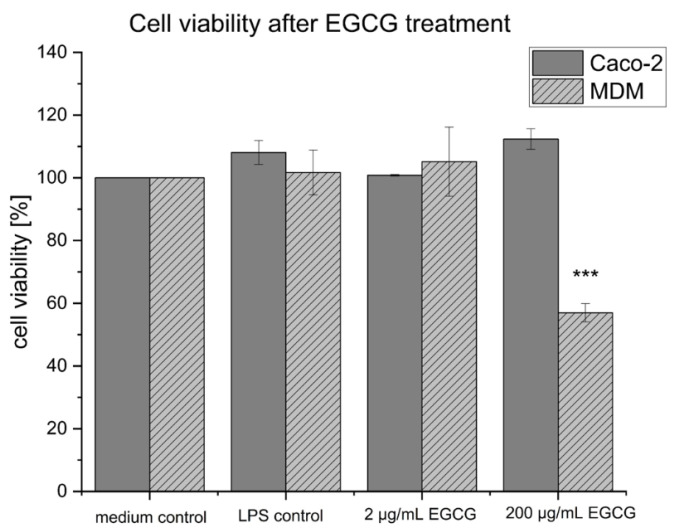
Cell viability of the Caco-2 cells and MDM after LPS stimulation and treatment with 2 µg/mL and 200 µg/mL EGCG compared to the medium control. Cell viability was determined by MTT assays at the end of the co-culture experiments. No cytotoxic effect was observed for the concentration of 2 µg/mL EGCG. A significant decrease in the cell viability compared to all other groups was measured for the MDM after the treatment with 200 µg/mL EGCG while the Caco-2 cells maintained full cell viability. Results are displayed as mean ± standard deviation for in summary *n* = 6 wells for each group based on two biological replicates. The *p*-value *** *p* < 0.001 imply significant differences.

The cell viability of the co-culture setup was addressed after performing the EGCG treatment of the inflamed co-culture. MTT assays were performed separately for the Caco-2 cells located in the apical compartment and the MDM located in the basolateral compartment of the co-culture setup. EGCG showed no cytotoxic effect on the Caco-2 cells, as the cell viability did not decrease compared to the medium control (Figure 3). For the MDM, EGCG in the concentration of 2 µg/mL resulted in no decrease in cell viability, while the concentration of 200 µg/mL led to a decrease in cell viability of the MDM to 57 ± 5.1%.

### 2.4. Effect of EGCG on Caco-2 Monolayer

**Figure 4:**
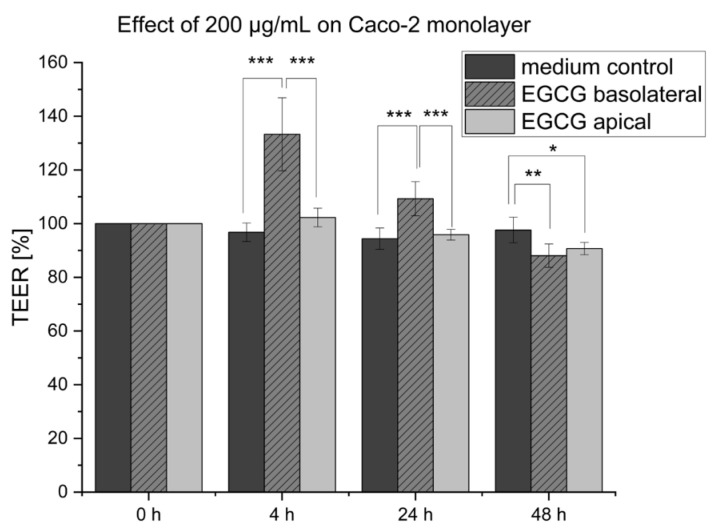
Effect of the stimulation of the Caco-2 monolayer with 200 µg/mL EGGC for 48 h either in the basolateral or apical compartment. The TEER value of the basolateral stimulated group increased significantly after 4 h of incubation. This effect was still measurable after 24 h; however, the TEER value decreased in comparison to the 4 h measurement. After 48 h, the TEER values of the basolateral stimulated Caco-2, and in addition, the apical-stimulated Caco-2 decreased in comparison to the medium control. Results are represented as mean ± SD for in summary *n* = 9 wells for each group performed in three biological replicates. * *p* < 0.05, ** *p* < 0.01, and *** *p* < 0.001 indicate significant difference.

The EGCG treatment in the concentration of 200 µg/mL showed a significant increase in the Caco-2 barrier integrity of the co-culture setup, and additionally, an effect on the cytokine release of the MDM was observed. To further analyze the effect of EGCG on the in vitro setup, the effect of EGCG only on the Caco-2 monolayer and without an inflammation stimulus was investigated. Figure 4 depicts the TEER values that were measured for 48 h after the stimulation with 200 µg/mL EGCG either in the basolateral or apical compartment. After 4 h of stimulation, a strong increase in the TEER values to 133.3 ± 10.2% of the basolateral treated cells in comparison to the medium control (96.8 ± 3.6%) was determined. This effect was still prominent after 24 h, with a decreased value of 109.3 ± 5.8% which was further reduced after 48 h (88.1 ± 5.0%). In contrast, the cells apically stimulated with EGCG showed no significant increase in TEER (102.3 ± 3.4%) in comparison to the medium control. The TEER value decreased slightly to 90.7 ± 2.5% after 48 h.

### 2.5. Comparison of EGCG’s Efficacy with IBD-Related Active Pharmaceutical Ingredients

In our previous work, we investigated the effect of commonly applied APIs related to the severity of the disease (from level 1 = minor inflammation to level 4 = major inflammation) using the same co-culture system and the same concentration of 200 µg/mL [23]. Table 1 compares the efficacy of EGCG with our results for the four APIs: 5-aminosalicylic acid (5-ASA, severity level 1), the corticosteroid Prednisolone (severity level 2), the immunosuppressant 6-Mercaptopurine (6-MP, severity level 3), and the monoclonal antibody Infliximab (severity level 4). EGCG showed barrier stabilizing effects after 24 h, similar to the immunosuppressant 6-Mercaptopurine (6-MP) and the biological drug Infliximab. Based on the effect of EGCG after 4 h of incubation, the result fits the maximal effect that was measured for 6-MP after 48 h. Referenced to the decrease in pro-inflammatory cytokine release of IL-6 and IL-8, EGCG corresponds to the effect of the corticosteroid Prednisolone.

## 3. Discussion

The effect of EGCG on the TEER values of the Caco-2 cells was investigated over 48 h. Two conditions were tested: the stimulation of the Caco-2 monolayer with 200 µg/mL EGCG in the apical or basolateral compartment and the investigation of EGCG as an anti-inflammatory compound for the treatment of the LPS-inflamed co-culture at the concentrations 2 µg/mL and 200 µg/mL. Both experiments showed a comparable response. The TEER values of the Caco-2 cells increased significantly for the basolateral stimulation of the monolayer (to 133.3 ± 10.2% after 4 h) and considerably elevated for the co-culture treatment with 200 µg/mL EGCG (to 165.7 ± 4.6% after 4 h). Hereafter, the effect decreased over time; however, the treated co-culture still regained the full barrier integrity after 48 h compared to the LPS and the medium control. Compared to the efficacies of APIs related to the severity level of IBD that we investigated in our previous study [23], the barrier stabilizing effect corresponds to the immunosuppressant 6-Mercaptorpurine (maximal effect: 134.33 ± 10.3% after 48 h) and the monoclonal antibody Infliximab (92.2 ± 6.2% after 24 h), representing level 3 and level 4 of severity. In the literature, Ran et al. and Oz et al. both observed analogous effects of EGCG and sulfasalazine in IBD mouse models [12,13]. However, there is a lack of data for the application of EGCG with IBD-related APIs. The results of our study confirm that EGCG has the potential to be a complementary medicine for IBD patients and should be further investigated. The decreasing effect of EGCG can be correlated with the auto-oxidation of EGCG in the cell culture medium. In the literature, the investigation of EGCG stability in 200 mM phosphate buffered saline at 37 °C showed the degradation into EGCG auto-oxidation products (EAOPs). After a mere 4 h of incubation, EGCG was undetected by HPLC analysis, whereas the content of the EGCG component gallic acid (GA) increased [17]. Therefore, the enhancement of the stability of EGCG and the prevention of auto-oxidation might play a key role in the application of EGCG as complementary medicine in vivo.

Focusing on the barrier stabilizing effect in more detail, Watson et al. observed the prevention of epithelial barrier dysfunction induced by INF-γ stimulation of the epithelial cell line T84 either by pre-treatment with 100 µM EGCG or simultaneous treatment with 100 µM EGCG and stimulation with 20 ng/mL INF-γ [24]. This underlines the positive effect of EGCG on the barrier integrity that was observed in our study using 200 µg/mL (approximately 0.44 mM) EGCG. Additionally, a strong effect on TEER values of Caco-2 cells as monolayer was observed by Amasheh et al. after the application of 200 µmol/L of quercetin, a further polyphenol and nutrition factor. The barrier integrity already increased in their experiments after a few hours of incubation, comparable to our EGCG experiments, while the highest TEER value of 157 ± 4% was measured after 48 h. Following that, the effect decreased; however, it was still higher for the 72 h measurement than for the control group [25]. In a previous study, Amasheh et al. showed that the increase in TEER after the treatment with quercetin may be connected to an increase in the barrier-forming tight junction (TJ) protein claudin-4 expression and the assembling of claudin 4 in TJ domains and subdomains [26]. The alternation of the TJ network is also a conceivable effect of EGCG on barrier integrity. The restoration of the TJ-proteins zonula occludens-1 (ZO-1), occludin, and claudin-1 after an intestinal injury induced by cyclophosphamide after EGCG treatment was recently shown in mice [27]. Different IBD-related animal studies prove the positive effect of EGCG on DAI, histological damage, and intestinal permeability [11,12,13,28,29]. Furthermore, the potential of EGCG-rich Polyphenol E was investigated in a human pilot study, where UC patients received this medication as complementary medicine to their conventional drugs compared to a placebo group. A total of 66.7% of the patients in the EGCG-rich Polyphenol E group responded to treatment. Moreover, every patient that responded to the complementary treatment showed an improvement in their endoscopic score [30]. Despite that, the full mechanisms behind the barrier-stabilizing effect of EGCG have not been fully investigated yet.

EGCG has not only the potential to improve barrier integrity, furthermore it is reported to have immunomodulating properties [10]. In our in vitro assay, we aimed for the simulation of IBD, and therefore, we integrated MDM as a component of the immune system, which enables the investigation of pro-inflammatory cytokines. We observed no significant effect for the treatment with 2 µg/mL EGCG, although we detected a significant decrease in the release of IL-8 back to the level of the healthy co-culture after the treatment with 200 µg/mL EGCG. Additionally, IL-6 was undetected after the treatment with 200 µg/mL EGCG. The release of the anti-inflammatory cytokine IL-10 was not significantly affected, and for TNF-α, no significant change was measured. However, especially for the TNF-α measurements, a high standard derivation was present based on the donor dependency of the MDM. Nevertheless, the human origin and the potential to release high amounts of different cytokines induced by LPS stimulation [31] are advantageous for simulating IBD. In comparison to the APIs for the treatment of IBD, EGCG showed a similar decrease in the release of IL-6 (to 0%) and IL-8 (to 14.2%) to the glucocorticoid (GC) Prednisolone (level 2 of severity). The mechanism of action of Prednisolone includes the binding to the glucocorticoid receptor (GR) followed by an interaction of the GC with the GR, leading to changes in the conformation and the translocation from the cytoplasm to the nucleus. Subsequently, the GC/GR complex binds to specific glucocorticoid-responsive elements (GREs), which stimulate and suppress gene transcriptions, including the synthesis of NF-κB and pro-inflammatory cytokines [32,33,34]. The activation of NF- κB and its translocation into the nucleus play a central role in IBD [35]. The mode of action of EGCG has not been fully investigated yet; however, effects on different pathways were identified in previous studies [36]. For instance, Yang et al. showed that EGCG successfully inhibited the IκB kinase complex activity and, therefore, the activation of NF- κB in vitro [37]. The blocking of NF- κB activation leads to a reduced release of numerous pro-inflammatory cytokines [35]. Shin et al. showed that the treatment of inflamed human mast cells-1 (HMC-1) with 100 µM EGCG resulted in a decrease in the TNF-α, IL-6, and IL-8 level by the attenuation of NF- κB and the extracellular signal-regulated kinase (ERK) [38]. Additionally, these effects on the release of pro-inflammatory cytokines have been proven by numerous animal studies [11,12,13]. In regard to human-derived data, LPS-stimulated CD14^+^ macrophages alone mixed with the T cell subpopulation CD4^+^ CD45^+^ RO, isolated from the peripheral blood of IBD patients, showed a decrease in pro-inflammatory cytokine production as well as a significantly induced apoptosis of the mentioned cell populations after 24 h of treatment with 5 µg/mL EGCG [39]. Correlating with these results, we observed in the co-culture system a significant decrease in the MDM viability to 57 ± 5.1% after 48 h of treatment, while the Caco-2 cells were not affected. However, to investigate the cell viability in more detail and to increase the reliability, additional methods such as the adenylate kinase assay might be considered.

One key factor for high efficacy and bioavailability in vivo is the stability of EGCG. The auto-oxidation in vitro is reported as lower when higher initial concentrations of EGCG were applied [17,40,41]. Additionally, several factors that have an influence were identified, such as storage conditions, pH, temperature, or the level of serum albumin in the blood plasma [16,18]. In human blood plasma, the peak concentration for an oral dose of EGGC (2 mg/kg) taken after overnight fasting was reached after 1 to 2 h and was decreased constantly to undetectable amounts after 24 h. The calculated elimination half-life of EGCG was 3.4 ± 0.3 h [42]. In our in vitro experiments, we observed the most prominent effect of EGCG after 4 h of treatment. Further open questions are the dose-finding and the time and frequency of administration [16]. Based on our results, EGCG should be administered daily. However, the transfer of dosages from in vitro to in vivo remains a significant hurdle. It is reported that the oral application in vivo of an equivalent dosage of 10 µM to 100 µM in vitro in the form of 2-3 cups of green tea led to a plasma concentration of only 0.1 to 0.6 µM [43]. This underlines the need for increasing the bioavailability of EGCG in vivo. An improvement of the plasma level in humans was obtained by the intake of EGCG without caffeine yet in combination with the antioxidant ascorbic acid and omega-3 fatty acids derived from salmon after an overnight fasting period [16]. Additionally, formulation strategies to attenuate the auto-oxidation processes might be a conceivable way to improve bioavailability. For instance, the stabilization of EGCG by encapsulation in chitosan nanoparticles resulted in enhanced intestinal absorption in mice and, therefore, higher bioavailability [44]. Recently, Wang et al. improved the storage stability by forming an inclusion complex of EGCG and γ-cyclodextrin while combining the therapeutical potential of both compounds [15]. Parallel to the susceptibility for auto-oxidation, it is shown that tea catechins similar to EGCG undergo biotransformation such as methylation, glucuronidation, sulfation, and ring-fission metabolism depending on the tissue and the species, leading to catechin metabolites with unknown biological activities [19].

The understanding of the metabolic mechanisms and the investigation of the bioavailability, efficacy, and safety of the metabolites are further key factors in evaluating the potential of natural products such as tea catechins. The here presented in vitro model provides the possibility to test the efficacy and cytotoxicity of improved EGCG formulation as well as identified natural metabolites related to IBD. A key factor for the usage of EGCG is the further assessment of the current bottlenecks, such as dose-finding, investigation of biotransformation, and bioavailability in vivo to benefit from the full potential of EGCG as complementary medicine. On this occasion, the application as complementary medicine is not only limited to IBD but can be beneficial for various diseases with uncontrolled immune activation, such as multiple sclerosis, psoriasis, and rheumatoid arthritis [9]. The amelioration of inflammatory processes and immune responses, as well as oxidative stress by natural dietary polyphenols, could assist in the prevention of carcinogenesis for colorectal cancer [45], which is known as the long-term complication of IBD [46]. EGCG itself is reported as an epigenetic regulator for cancer and a strong chemoprotective compound that interferes with different cancer signaling pathways [21,47]. Furthermore, the potential of EGCG for the treatment of respiratory diseases such as acute respiratory distress symptom (ARDS) or COVID-19 due to its antioxidant, anti-fibrotic properties and the ability to attenuate the production of various inflammatory mediators is discussed in the recent literature [48,49].

## 4. Materials and Methods

### 4.1. Cell Culture Experiments

#### 4.1.1. Co-Culture Setup

The effect of EGCG as CAM for the treatment of intestinal inflammations was tested on an epithelium (Caco-2)/immune cell (monocyte-derived macrophages (MDM)) co-culture, as described in our previous work [23]. In brief, the Caco-2 cells were seeded in the apical compartment of Transwell^®^ inserts (12-well plate, 12 mm Transwell^®^ with polyester membrane inserts, 0.4 µm pore size, 1.12 cm^2^ growth area, Corning, Glendale, AZ, USA) until an intact epithelial barrier with TEER values > 500 Ω* cm^2^ was established. Primary monocytes were isolated from human blood, obtained from the Blutspendezentrale Saar-Pfalz GmbH (Saarbrücken, Germany), and differentiated by the addition of granulocyte-macrophage colony-stimulating factor (GM-CSF, Gibco, Thermo Fisher Scientific, Waltham, MA, USA) into MDM. After 24 h of differentiation, the MDM were washed, and a fresh cell medium with GM-CSF was added. Subsequently, the MDM were incubated for 72 h and then seeded in the basolateral compartment of a 12-well plate. The MDM were grown in the well plate for 24 h, washed, and the Transwell^®^-inserts with the Caco-2 cells were carefully placed above the macrophages. The barrier integrity of the co-culture was investigated by TEER measurements (see Section 4.2.1 TEER measurements). All cells, single monolayers, and the co-culture setup were maintained at a constant humidity with 5% CO_2_ at 37 °C.

#### 4.1.2. Inflammation of Co-Culture

To simulate the inflammatory state of IBD, the co-culture was inflamed by using Lipopolysaccharides (LPS) from *E. coli* (Sigma-Aldrich, Merck Millipore, Darmstadt, Germany) in a final concentration of 200 ng/mL for each well (12-well plate, 12 mm Transwell^®^ with polyester membrane inserts, 0.4 µm pore size, 1.12 cm^2^ growth area, Corning, Glendale, AZ, USA). The LPS was added to the basolateral compartment directly after starting the co-culture in all wells except for the medium control, which served as negative control (no inflammation; healthy state).

#### 4.1.3. ECGC Treatment of Inflamed Co-Culture

EGCG (Viktoria Apotheke, Saarbrücken, Germany) was applied to the inflamed co-culture in two concentrations, 200 µg/mL and 2 µg/mL, on the inflamed co-culture to investigate a potential anti-inflammatory effect. EGCG was added to the basolateral compartment containing the MDM directly after the LPS stimulation, except for the wells with the medium control and the LPS control, which was defined as a positive control (no treatment). The TEER values were measured after 4 h, 24 h, and 48 h of incubation to analyze the EGCG effect on the epithelial barrier properties. After 48 h of incubation, the supernatant of the basolateral compartment was collected, and the release of different cytokines was measured by enzyme-linked immunosorbent assay (ELISA).

#### 4.1.4. Investigation of Cell Viability of the Co-Culture Setup after EGCG Treatment

The cell viability after the incubation with 200 µg/mL and 2 µg/mL of EGCG was analyzed directly at the end of the treatment experiments by performing individual MTT assays for the Caco-2 located in the apical and the MDM located in the basolateral compartment.

#### 4.1.5. Investigation of ECGC Effect on Caco-2 Monolayer

The potential effect of EGCG on the integrity of the intestinal barrier was investigated utilizing only the Caco-2 monolayer cultivated in the apical compartment of a Transwell^®^ system without an inflammatory stimulus. The 200 µg/mL and 2 µg/mL EGCG were applied to the Transwell^®^ plate in the basolateral compartment (simulating the blood side) or in the apical compartment (simulating the lumen side). TEER values were measured after 4 h, 24 h, and 48 h.

### 4.2. Analytical Methods

#### 4.2.1. TEER Measurements

The TEER values of the Caco-2 cells in the Transwell^®^ inserts were measured on a heating plate (W10, VWR, Germany) at 37 °C with the Epithelial Voltohmmeter EVOM2 (World Precision Instruments, Friedberg, Germany) and the STX2 Chopstick Electrode Set (World Precision Instruments, Friedberg, Germany). Before the measurements, the Chopstick Electrode set was cleaned in 70% 2-propanol (Th. Geyer, Renningen, Germany) for 10 min and dried at room temperature (RT).

#### 4.2.2. ELISA Measurements

For performing ELISA to measure the cytokine release by the MDM in the co-culture system, the Human Uncoated ELISA Kits for the tested cytokines (TNF-α, IL-6, IL-8, and IL-10) from Thermo Fisher Scientific were used and carried out according to the provided protocol. In brief, the coating of 96-well plates (Corning, Glendale, AZ, USA) with the specific capture antibody followed by incubation at 4 °C overnight was performed in the first step. After washing the wells three times with wash buffer (1x DPBS (Gibco, Thermo Fisher Scientific, Waltham, MA, USA) with 0.05% Tween-20 (Merck Millipore, Darmstadt, Germany)), the wells were blocked with 1x ELISA Spot Diluent and incubated at RT for one hour. The samples were prepared by centrifugation for five minutes at 1000× *g*. The following dilutions of the samples in MDM medium were transferred to the 96-well plates: 1:10 and 1:100 for the TNF-α ELISA; not diluted and 1:10 for the IL-6 ELISA; 1:100 and 1:1000 for the IL-8 ELISA; not diluted and 1:2 for the IL-10 ELISA. 100 µL of each sample was applied to the wells. The provided standard for each cytokine was reconstituted and transferred to the plates according to the protocol. The plates were stored at 4 °C overnight. Subsequently, the wells were washed five times with wash buffer, 100 µL detection antibody was added in each well, and the plates were incubated for one hour at RT. After that, the wells were washed again five times. The 100 µL/well Avidin–horseradish peroxidase (HRP) was added, and the plates were incubated for 30 min at RT. All wells were washed six times with wash buffer, and 100 µL/well 3, 3′, 5, 5-tetramethylbenzidine (TMB)-substrate was applied, followed by incubation for 15 min at RT protected from light. In the end, 100 µL/well 1 M ortho-phosphoric acid (stop solution) was added to stop the enzymatic reactions. The absorbance of the samples was measured at the wavelength 450 nm with the Plate Reader Synergy2 (BioTek Instruments GmbH, Bad Friedrichshall, Germany), and background correction was performed by subtraction of the 570 nm reading. The concentration of each sample was calculated based on the respective calibration curve.

#### 4.2.3. MTT Assay

To investigate the cell viability after the stimulation of the MDM and Caco-2 co-culture, MTT assays were performed at the end of the experiment. The cell viability was calculated individually for the MDM and the Caco-2 cells in the co-culture setup. The MTT assay was performed as described by Scherließ et al. with some adjustments due to the Transwell^®^ system [50]. In brief: at the end of the co-culture experiment (see section Treatment with EGCG), the cells were washed twice with 500 µL (Caco-2) and 1000 µL (MDM) 1x HBSS (Gibco, Thermo Fisher Scientific, Waltham, MA, USA). The medium control wells were used as a positive control (100% viability), and 1% Triton-X-100 (AppliChem GmbH, Darmstadt, Germany) was used as negative control (0% viability). Subsequently, 0.5 mg/mL MTT reagent ((3-(4,5-dimethylthiazol-2-yl)-2,5-diphenyl tetrazolium bromide, Agros Organics, Thermo Fisher Scientific, Waltham, MA, USA) was added in a volume of 500 µL (apical) and 750 µL (basolateral). The cells were incubated for 4 h at 37 °C under permanent shaking at 35 rpm and exclusion of light. After removal of the MTT reagent, 500 µL apical and 750 µL basolateral DMSO were added to each well and incubated for 15 min at 37 °C under permanent shaking at 35 rpm and exclusion of light. The absorbance was measured at the wavelength 550 nm with the Plate Reader Synergy2 (BioTek Instruments GmbH, Bad Friedrichshall, Germany). The cell viability was calculated using the following formula:

(1) $$\mathrm{Viability}\;[\%]=\frac{{\mathrm{absorbance}}_{\mathrm{test formulation}}-{\mathrm{absorbance}}_{\mathrm{Triton X}-100}}{{\mathrm{absorbance}}_{\mathrm{medium control}}-{\mathrm{absorbance}}_{\mathrm{Triton X}-100}}*100$$

Formula (1) indicates the calculation of cell viability in %.

### 4.3. Statistical Analysis

The data are presented as mean ± standard deviation (SD) based on the performance of independent replicates in different numbers of repetitions (n). For the statistical analysis, the program OriginPro 2021 was used. When two groups were compared, a two sampled-t-test was performed; for the comparison of > two groups, one-way analysis of variance (ANOVA) and the Bonferroni test as a post hoc test were applied.

## 5. Conclusions

Following the investigation of the efficacy of EGCG in our human cell-based co-culture consisting of epithelial and immune cells, it is possible to conclude that EGCG is a potential complementary medicine for IBD patients. The treatment with EGCG at a concentration of 200 µg/mL showed a positive effect on the barrier integrity of the Caco-2 cells and the release of IL-6 and IL-8 by the MDM. Compared to APIs that are commonly applied in IBD, EGCG showed comparable barrier-stabilizing effects to the immunosuppressant 6-MP and the monoclonal antibody Infliximab. Furthermore, the attenuation of the IL-6 and IL-8 release was similar to the corticosteroid Prednisolone. The strongest effect on the TEER values was observed after 4 h of incubation. Hereafter, the effect decreased over time; however, the barrier integrity in the co-culture system was restored and intact according to the TEER, even after 48 h of incubation. The decreasing effect can be associated with the auto-oxidation and low stability of EGCG, which is reported as the main challenge for the transfer of in vitro data to the in vivo situation. Moreover, the biological effect and toxicity of the metabolites need to be critically investigated. Due to the human origin of the cells and the usage of primary donor-dependent immune cells, we could analyze the effect of EGCG addressing the human species; however, the processes of biotransformation of EGCG in vitro and in vivo should be addressed in further studies. In clinical trials, the efficacy of EGCG is often limited, and only low plasma levels have been achieved. To improve the stability and bioavailability of EGCG in the future, formulation strategies such as stabilizing by encapsulation in nanoparticles, conjugation to other compounds, or the application of additional substances such as ascorbic acid as antioxidants are conceivable. Once the bottlenecks have been mitigated, the opportunities for EGCG exceed usage as a complementary medicine for IBD, other autoimmune diseases as well as a cancer treatment due to the numerous positive properties.

## Figures and Tables

**Table 1:** Comparison of the efficacy of EGCG and IBD-related APIs that are utilized depending on the severity level of the disease (level 1 = minor inflammation up to level 4 = major inflammation). All compounds were applied in a concentration of 200 µg/mL to the co-culture system. ↓, ↓↓ and ↓↓↓ represent a significant difference of *p* < 0.5, *p* < 0.01, and *p* < 0.001. Abbreviations: 5-ASA = 5-aminosalicylic acid; 6-MP = 6-Mercaptopurine.

API	TEER in % after 24 h	TNF-α	IL-6	IL-8
5-ASA (level 1)	68.23 ± 7.2%	-	-	↓
Prednisolone (level 2)	81.29 ± 13.1%	-	↓↓	↓↓↓
6-MP (level 3)	106.73 ± 6.7% maximal effect after 48 h (134.33 ± 10.3%)	-	-	-
Infliximab (level 4)	92.2 ± 6.2%	↓	-	↓↓
EGCG	93.47 ± 14.8% maximal effect after 4 h (165.72 ± 4.6%)	-	↓↓	↓↓↓

## Data Availability

Data are contained within the article.
